# Recessive Mutations in *POLR3B* Encoding RNA Polymerase III Subunit Causing Diffuse Hypomyelination in Patients with 4H Leukodystrophy with Polymicrogyria and Cataracts

**DOI:** 10.1007/s00062-015-0472-1

**Published:** 2015-10-19

**Authors:** E. Jurkiewicz, D. Dunin-Wąsowicz, D. Gieruszczak-Białek, K. Malczyk, K. Guerrero, M. Gutierrez, L. Tran, G. Bernard

**Affiliations:** 1 Department of Diagnostic Imaging, The Children’s Memorial Health Institute, Al. Dzieci Polskich 20, 04-730 Warsaw, Poland; 2 Neurology and Epileptology Department, The Children’s Memorial Health Institute, Al. Dzieci Polskich 20, 04-730 Warsaw, Poland; 3 Department of Medical Genetics, The Children’s Memorial Health Institute, Al. Dzieci Polskich 20, 04-730 Warsaw, Poland; 4 Department of Paediatrics, The Medical University of Warsaw, Dzialdowska1, 01-184 Warsaw, Poland; 5 Pediatric Neurodegenerative Laboratory, Departments of Neurology and Neurosurgery and Pediatrics, McGill University Health Center Research Institute, McGill Universit, Montreal, Quebec, Canada

**Keywords:** Hypomyelination, 4H leukodystrophy, Magnetic resonance imaging, Children, Polymicrogyria, Cataract

## Abstract

The diagnosis of 4H leukodystrophy (hypomyelination, hypogonadotropic hypogonadism, and hypodontia) is based on clinical findings and magnetic resonance imaging (MRI). Recently, mutations of the genes encoding Pol III (RNA polymerase III) subunit A (POLR3A) and subunit B (POL3B) have been identified as the genetic causes of hypomyelination. We describe two Polish female siblings aged 5 and 10 years with compound heterozygous mutations in POLR3B. They both presented with similar clinical symptoms and MRI findings presenting as 4H leukodystrophy, and the association of polymicrogyria and cataract. According to our observation in young children with the absence of hypogonadotropic hypogonadism, brain MRI pattern is very essential in proper early diagnosis of 4H leukodystrophy. All clinical and radiological results are of course helpful, however genetic conformation is always necessary.

## Introduction

Hypomyelination, hypogonadotropic hypogonadism, and hypodontia is an autosomal recessive hypomyelinating disorder first reported in children and adults by Wolf et al. and Timmons et al. [1, 2]. These patients had a hypomyelinating leukodystrophy with prominent cerebellar features and followed a progressive course. At that time, no molecular cause was identified. In 2006, Timmons et al. [2] suggested the term “4 H syndrome”, although it should be noted that the non-neurological features hypodontia and/or hypogonadism are not always present [2, 3].

Several syndromes causing diffuse hypomyelination have been documented, including hypomyelination, hypodontia, and hypogonadotropic hypogonadism (4H) syndrome (MIM 612440) [1, 2, 4], hypomyelination with atrophy of the basal ganglia and cerebellum (H-ABC) (MIM 612438) [5, 6], diffuse cerebral hypomyelination with cerebellar atrophy and hypoplasia of the corpus callosum [7, 8], tremor–ataxia with central hypomyelination [9], Pelizaeus–Merzbacher disease and Pelizaeus–Merzbacher-like syndrome [10, 11], among others.

The diagnosis of 4H syndrome is based on clinical findings and magnetic resonance imaging (MRI). Recently, mutations of the genes encoding POLR3 (RNA polymerase III) subunits POLR3A (*POLR3A*), subunit POLR3B (*POLR3B*) and POLR1C (*POLR1C)* have been identified as the genetic causes of this disorder [3, 8, 12–18]. POLR3 is an enzyme responsible for transcription of specific noncoding small RNAs involved in the regulation of essential cellular processes (transcription, RNA processing, translation) [19]. It is suggested that mutations in *POLR3A, POLR3B*, and *POLR1C* lead to abnormal POLR3 function and abnormal production of proteins important for development of the central nervous system white matter [18, 20].

In 2014, Wolf et al. published a multinational cross-sectional observational study on the clinical, molecular, and MRI characteristics of 105 patients with mutation-proven 4H leukodystrophy caused by mutations in *POLR3A* or *POLR3B*. A total of 62 patients had mutations in the *POLR3B* gene [21]. In our publication we report two Polish female siblings diagnosed and treated at the Children’s Memorial Health Institute in Warsaw, Poland, with compound heterozygous mutations in *POLR3B*. They both presented with compatible clinical and MRI features of 4H leukodystrophy, together with polymicrogyria (PMG) and cataracts, which have been never reported in 4H patients before.

## Methods

### Magnetic Resonance Imaging

Brain MRI was performed in both patients using a 1.5 T scanner with 8-channel phased-array head coil. TSE T2-weighted images in axial (TR/TE, 3930/108 ms), coronal (TR/TE, 5290–5450/135), and sagittal (TR/TE, 5500–5610/135–143 ms) planes, axial fl2D T1-weighted (TR/TE, 234/4.8 ms), and tfl3d_nsIR sag_iso (TI/TR/TE, 1100/1840/39 ms) were acquired. Diffusion-weighted 3-scan trace in the transverse plane by using echo-planar imaging (TI/TR/TE, 1100/6000/72 ms) was performed with b-values of b = 0. 500, and 1000. The MRI characteristics were analyzed, with particular attention paid to the structural anomalies and the state of brain myelination, according to previously published criteria [22–24].

Retrospectively, two neuroradiologists independently reviewed all the MR images. Discrepancies were solved by consensus. Sizes of the cerebellar hemispheres and vermis, myelination of the corpus callosum, posterior limb of the internal capsules (PLIC), cerebral and cerebellar white matter, and optic radiation were evaluated. The sizes of patients’ cerebellum were subjectively compared with the cerebellum of aged-matched controls. Cerebral atrophy was defined as volume loss leading to enlargement of the ventricles and subarachnoid spaces. Cerebellar atrophy was assessed by evaluating the degree of enlargement of the fissures of the cerebellar hemispheres and vermis. White matter hypomyelination was assessed on axial T1 and T2-weighted images as previously defined [22–24].

### DNA Screening

Peripheral blood samples were obtained from siblings and their parents after obtaining informed consent. Genomic DNA was extracted using standard methods. Mutation analysis was first performed in Montreal, Canada as part of a REB (Research Ethics Board)-approved research project using whole exome sequencing, followed by Sanger sequencing for validation of variants and segregation analysis. The results were confirmed in a clinical lab—Medgen in Warsaw, Poland.

## Case Report

### Case 1

This patient was admitted for the first time to our hospital at the age of 8 years for investigations of neurological abnormalities and dysmorphic features. Her parents are healthy. The perinatal history revealed that she was born after an uneventful second pregnancy and delivery. Of note, the patient’s head circumference at birth was 31 cm (< 3rd percentile). Hypotonia and bilateral hip dysplasia were noted in the neonatal period. Hip dysplasia was surgically corrected at the age of 2 years and the girl was able to walk 10 months later despite the right leg being 2 cm shorter than the left. Tremors in the extremities and trunk occurred at the age of 30 months and were more intense in the morning. Over time, gait disturbances and cerebellar signs became more pronounced. Intellectual disability was diagnosed at the age of 4 years (IQ 34). She had vision problems and binocular cataracts were diagnosed at the age of 7.5 years. Cataracts progressed with such intensity that ophthalmological surgery was necessary 6 months later. Myopia was also noted. She lost the ability to walk at the age of 8 years. Microcephaly (head circumference 48 cm—below 3rd percentile) was also noted at the age of 8 years. At her most recent visit with us, the patient was 12-year-old. She was still axially hypotonic, wheelchair bound, anarthric, G-tube fed because of dysphagia, had severe cerebellar features and only mild pyramidal features. No epileptic seizure was observed clinically, but her electroencephalography (EEG) revealed epileptic discharges during sleep. All clinical features are summarized in Table 1.

**Table 1:** Clinical symptoms and signs in female siblings with 4H syndrome

Type of symptoms and signs	Case 1 age of onset		Case 2 age of onset	
Cataract	+	Diagnosed and operated at the age of 8 years	+	Diagnosed and operated at the age of 3 years
Myopia	+	8 years	+	3 years
Nystagmus	−	−	−	−
Optic atrophy	−	−	−	−
Sensorineural hearing loss	+ Right ear	3 years	+ Left ear	3 years
Microcephaly (below 3rd percentile)	+	At birth	+	At birth
Dysphagia	+	4 years	−	−
Hypotonia	+	At birth	+	At birth
Brachial plexus paresis	−	−	+ Left	At birth
Tremors	+	2.5 years	+	2.5 years
Ataxia	+	2 years	+	2 years
Dysmetria	+	3 years	+	3 years
Dysarthria	+	6 years	−	−
Developmental delay	+	1 year	+	1 year
Intellectual disability	+	4 years	+	4 years
Wheelchair use	+	8 years	−	−
Epileptic seizures	−	−	−	−
Epileptic discharges in EEG	+	3 years	+	3 years
Dysplasia of the hips	+	At birth operated at the age of 2 year	−	−
Flat -valgus feet	+	3 years	+	3 years
Short stature (below 3rd percentile)	+	2 years	+	2 years
Hypodontia	+	2 years	+	2 years
Hypogonadotropic hypogonadism	Too young	−	Too young	−

Genetic and metabolic tests were pursued due to the presence of dysmorphic features (deep set eyes, hypodontia, microcephaly) and developmental delay. Karyotype was normal (46XX), Rett syndrome, Angelman syndrome, Pelizaeus–Merzbacher disease, Krabbe disease, neuronal ceroid-lipofuscinosis, metachromatic leukodystrophy, and GM1/GM2 gangliosidosis were excluded and no other metabolic diseases were found. The patient’s body weight and height were reduced, although growth hormone deficiency, hypothyroidism, and adrenal insufficiency were excluded.

**Fig. 1:**
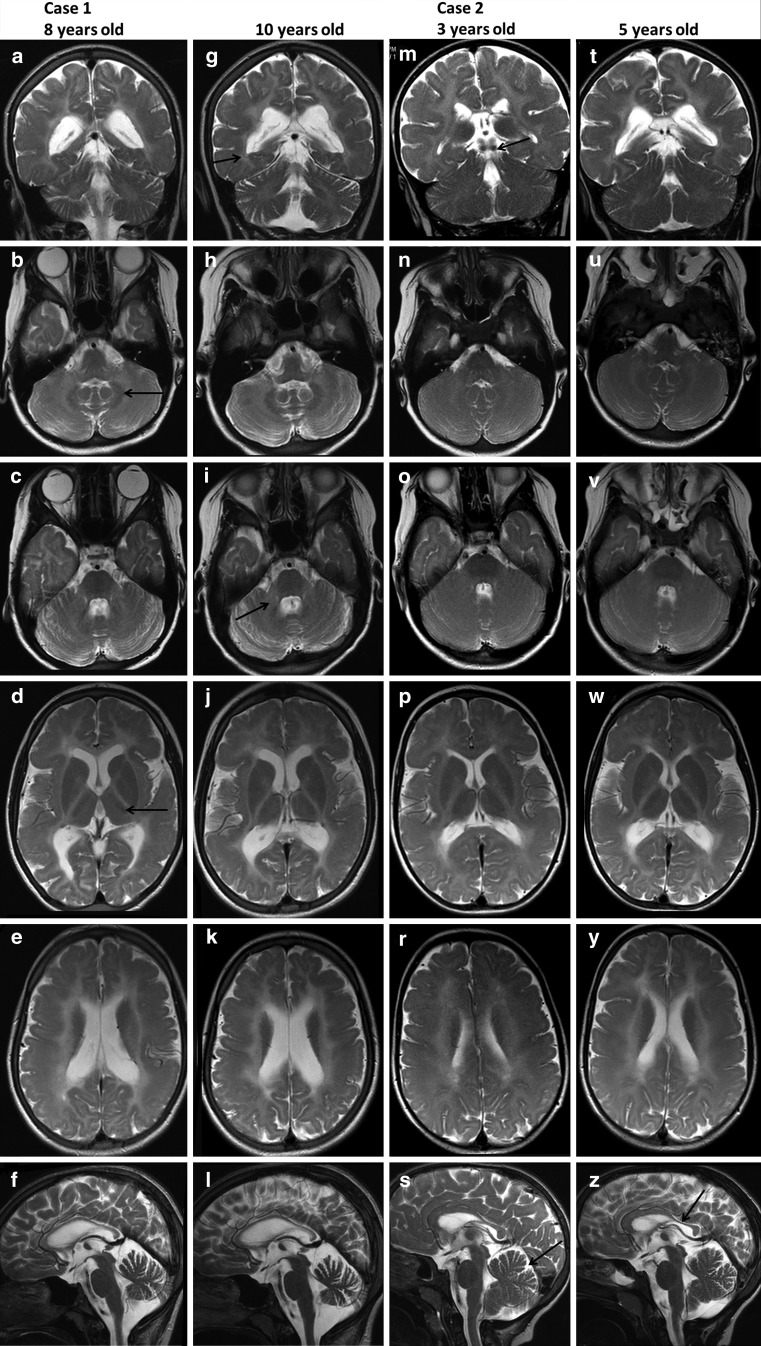
Brain magnetic resonance imaging (MRI) of the patients. T2-weighted images. Case 1 is seen in *A–L*. The first column (*A*–*F*) shows the brain MRI at the age of 8 years while the second column (*G–L*)—the MRI at the age of 10 years. Case 2 is shown in *M–Z*. The third column (*M–S*) shows the brain MRI at the age of 3 years while the fourth column (*T–Z*) shows the MRI—at the age of 5 years. Coronal T2-weighted images demonstrated very thin and slightly myelinated optic radiation (*arrow on G*) and myelinated inferior colliculi (*arrow on M*). Typical hypointensity of the dentate nucleus is seen on axial T2-weighted images (*arrow on B*) more evident in the older girl (*B, H*). Note the hyperintense signal of the middle cerebellar peduncle (*arrow on I*) demonstrating hypomyelination of this structure. In the older girl axial images (*C, I*) mild atrophy of cerebellar hemispheres is seen with little progression over 2 years. Cerebellar hemispheres did not show atrophy in the younger girl (*N, O, U, V*). Diffuse hyperintensity of the cerebral white matter is seen on supratentorial axial images of the brain. Ventroanterolateral nucleus of the thalamus appeared hypointense (*arrow, picture D*). Posterior limbs of internal capsules are not myelinated. Mild enlargement of the lateral ventricles is visible. Corpus callosum is thin and unmyelinated on midline sagittal T2-weighted images of the older girl (*F, L*). Note atrophy of the vermis. Slight progression of the atrophy of the vermis is seen. Corpus callosum of the younger girl (*S, Z*) is also unmyelinated and thinned posteriorly (isthmus and splenium); slight progression of changes. Note prominent, but within normal limits, primary fissure of the vermis (*arrow, picture Z*), which remained unchanged over 2 years

**Fig. 2:**
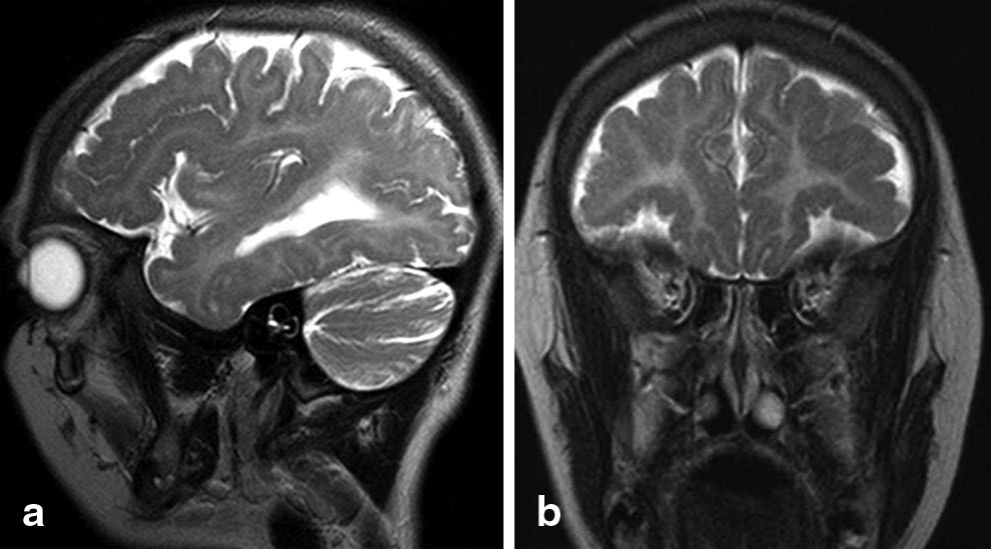
Sagittal (**a)**, and coronal (**b)** T2-weighted images of case 1 showing irregularity of the frontal cortex with shallow sulci and numerous small gyri, consistent with polymicrogyria

The cerebral MRI performed at the age of 8 and 10 years revealed diffuse supra- and infratentorial hypomyelination. Indeed, T2 hyperintensity of the white matter was observed in the cerebral and cerebellar hemispheres, as well as in bilateral middle cerebellar peduncles. The inferior colliculi were myelinated and visible as low signal intensity on T2- and high signal on T1-weighted images. Hypointense signal of the dentate nuclei was noted, indicating relatively preserved myelination. Mild cerebellar atrophy involved both the vermis and the hemispheres. The corpus callosum was thin and unmyelinated. Relatively, preserved myelination of the anterolateral thalamus and optic radiation was also observed. No hypointense signal was visible in the PLIC, indicating that the pyramidal tracts were not myelinated (Fig. 1). Mild enlargement of the lateral ventricles was observed. Additionally, diffuse bilateral and symmetrical frontoparietal PMG was noticed (Fig. 2). The signal of basal ganglia and brainstem was normal. At the time of the examinations, the patient was prepubescent and a pituitary gland of 5.5 × 2.5 × 9 mm was visualized (small for her age; pituitary gland diameter was compared with standards published by Fink et al. [25]). There was no hypophysis structural anomaly. Magnetic resonance findings are summarized in Table 2. During the 2 years of observation (two MRI examinations) a slight progression of atrophy involving the vermis and cerebellar hemispheres was observed. No change in the diffuse hypomyelination of the white matter was noted.

**Table 2:** Summary of the magnetic resonance findings

Magnetic resonance imaging	Case 1/at the age of 10 year	Case 2/at the age of 5 year
Hypomyelination of the cerebellar white matter	Yes	Yes less accentuated
Hypomyelination of middle cerebellar peduncles	Yes	Yes less accentuated
Atrophy of the cerebellar vermis	Yes	No
Atrophy of the cerebellar hemispheres	Yes mild	No
Myelinated dentate nuclei	Yes	Yes less accentuated
Atrophy of the cerebral hemispheres	Yes mild	No
Hypomyelination of the cerebral white matter	Yes	Yes
Myelinated optic radiation	Yes	Yes
Myelinated inferior colliculi	Yes	Yes
T2-hypointense posterior limb of the internal capsules signal	No	No
Corpus callosum unmyelinated	Yes	Yes
Thin corpus callosum	Yes	Yes/partially thin the splenium and isthmus
Polymicrogyria	Yes	Yes
Enlargement of the lateral ventricles	Yes	Yes only trigonum
Basal ganglia atrophy	No	No
Brain stem atrophy	No	No
Pituitary gland^a^ Anterior-posterior/high/lateral in mm	5.5 × 2.6 × 9 below normal values	5.6 × 3.9 × 9 in the borderline range

^a^Girls before puberty

### Case 2

The first patient’s younger sister was admitted at the age of 3 years. Her prenatal and perinatal histories were uneventful. In the neonatal period, she demonstrated clinical signs of a left brachial plexus paresis (Erb’s paresis). Both hips were also dysplastic and treated conservatively with orthopaedic instruments; orthopaedic surgery was not necessary. She started to walk without help at the age of 27 months. The girl was also hypotonic. The child was diagnosed with myopia and she developed cataracts very rapidly and at an earlier age than her sister. She needed ophthalmological surgery at the age of 3 years. Her dysmorphic features and other clinical symptoms were almost the same as those of her sister, including severe morning tremors, ataxic gait, and intellectual disability (IQ 50 at the age of 4 years). Speech development was also delayed: at the age of 6 years she was only able to say 20 words, but without dysarthria. Due to the similar clinical picture, neuroimaging was performed and a genetic etiology was suspected. Like her sister, she had microcephaly at birth, with a head circumference of 32 cm (< 3rd percentile), and at the age 3 years, with a head circumference of 44 cm (< 3rd percentile). Clinical features are summarized in Table 1.

MRI of the brain performed at ages of 3 and 5 years showed less pronounced hypomyelination in both infra- and supratentorial regions relative to her sister. Hypomyelination of the cerebellar white matter was less significant. The inferior colliculi were myelinated and visible as low signal intensity on T2- and high signal intensity on T1-weighted images. A slight hypointense signal of the dentate nucleus is evident (indicating relatively preserved myelination). The size of the cerebellar hemispheres and vermis were within normal limits, the primary fissure in the vermis was visible. The posterior part of the unmyelinated corpus callosum (isthmus and splenium) was thinned. Relatively, preserved myelination of the anterolateral thalamus and the optic radiation was observed. No signal hypointensity of the PLIC was noted, indicating that the pyramidal tracts were not myelinated, as in her sister (Fig. 1). Diffuse, bilateral, symmetrical frontoparietal PMG was also visualized. The basal ganglia and brainstem were within normal limits. At the time of examination the girl was prepubertal, and according to Fink AM et al. [25] her pituitary gland (5.6 × 3.9 × 9 mm) was borderline small. There was no hypophysis structural anomaly. Trigones of the lateral ventricles were slightly widened. MR findings are summarized in Table 2. Comparing the patient’s two examinations revealed mild atrophic progression in the isthmus of the corpus callosum.

## Molecular Results

Both patients were found to be compound heterozygotes for mutations in *POLR3B*: c.1939G > A (p.E647K) in exon 16 and c.2084-6A > G in intron 19. The mutation c.2084-6A > G has been previously reported and it is known to be disease-causing [3, 21]. Variant c.1939G > A (p.E647K) has not been reported in any database, nor in other POLR3-related leukodystrophy cases. *In silico* analysis using the bioinformatics tools MT (mutation taster), PP2 (Polyphen2), SIFT (Sorting Intolerant From Tolerant) and PROVEAN (Provean Variation Effect Analyzer) predicted this variant to be damaging. Segregation analysis revealed that the mother is a carrier of the intronic mutation and the father is a carrier of the c.1939G > A (p.E647K) variant. Whole exome sequencing analysis is still ongoing in order to identify the etiology of the PMG and cataracts as these clinical features are hypothesized to be caused by a mutation(s) in a second gene.

## Discussion

4H or POLR3-related leukodystrophy is inherited in an autosomal recessive fashion and is caused by mutations in one of three genes encoding RNA polymerase III (POLR3) subunits, that is, *POLR3A, POLR3B*, and *POLR1C* [8, 12–17]. Daoud et al. [3] noticed that POLR3A mutations are more frequent but a multinational cross-sectional observational report published in 2014 by Wolf et al. [21] concluded that patients from European backgrounds were more likely to have *POLR3B* mutations than other populations. The two patients we investigated with mutations in *POLR3B* genes showed diffuse hypomyelination of the cerebral and cerebellar white matter, hypodontia, and cataracts. Children were prepubertal, thus hypogonadotropic hypogonadism could not be assessed. Interestingly, pituitary volume in the older sister is below normal and in the younger sister is in the borderline small range [25]. All genetic, neuroimaging, and clinical features were characteristic for 4H leukodystrophy, except for PMG and cataracts. Mild atrophy of cerebellar hemispheres and vermis together with enlarged folia was observed in the older girl, while diffuse hypomyelination of cerebral white matter with unmyelinated corpus callosum were seen in both sisters. Such imaging findings are typical for 4H leukodystrophy and have been previously described in the literature [3, 17, 21].

In addition to the characteristics of 4H leukodystrophy, both patients presented bilateral frontoparietal PMG. PMG is a common cortical malformation characterized by an excessive number of abnormally small gyri. PMG may be uni- or bilateral, symmetrical or asymmetrical, focal or diffuse. Any region of the cerebral cortex can be affected. It can be caused by congenital infections, *in utero* ischemia or could be genetic in origin [26]. Several genes have been associated with PMG, including *GPR56, SRPX2, TUBB2B, TUBB3, PAX6, TBR2, KIAA1279, NHEJ1, RAB3GAP1, EOMES, COL18A1*, and *TUBA8* [26–29].

According to previously published data [3, 21, 31] myelination of the optic radiation was seen in almost all 4H patients (95 % of patients in a paper published by Wolf et al. [1] in 2014 [21]. We also noticed narrow, slightly T2-hypointense and poorly visible signal of the optic radiation. Relative T2 hypointensity of the anterolateral thalamus was seen in 91 % of the patients [21] and relative myelin preservation in the dentate nucleus was visualized in 93 % of the patients [20]; such features were also present in both our patients. We did not observe myelination of the corticospinal tracts at the level of the PLIC. Our results are in contrast to previously published papers, which reported hypointense dots in the PLIC. Focal myelination of the corticospinal tracts at the level of internal capsules was seen in 70 % of the patients with *POLR3B* mutations [3, 21, 31]. Mild atrophy of cerebral hemispheres, seen only in the older sister, was described by Wolf et al. [21] who had mentioned that supratentorial atrophy was rarely seen before the age of 10 years. We observed unmyelinated corpus callosum in both our patients with overall thinning in the older and partial thinning in the younger patient (splenium and isthmus). Thinned corpus callosum was found in all ten patients with *POLR3B* mutations in a paper published by Daoud et al. [3]. Wolf et al. [1] noted thinning of corpus callosum in all patients above 17 years of age, and concluded that among children below the age of 10 years thin corpus callosum is a more frequent finding in *POLR3A* than *POLR3B* mutations [21]. Mild atrophy of the vermis and cerebellar hemispheres were seen in the older patient while a deep primary fissure, although within normal limits, was noted in the younger patient. This is somewhat in contrast with Wolf et al. [21], who had found cerebellar atrophy in all *POLR3B (*except for one 3-year-old), although median age at last MRI examination among those patients was 10 years. Our investigations did not fully corroborate the observations published by Takanashi et al. [17] in a small group of patients concerning different patterns of cerebellar abnormalities and hypomyelination between *POLR3A* and *POLR3B* mutations. The authors found small vermis and cerebellar hemispheres with thin folia and enlarged fissures in all the patients with *POLR3B* mutations. They also suggested that patients with *POLR3B* mutations were affected by milder hypomyelination than those with *POLR3A* mutations. These observations stand in opposition to our findings, since our patients presented diffuse hypomyelination. The discrepancy in the size and atrophy of cerebellar hemispheres and vermis may be possibly explained by the ages of our patients (5 and 10 years), compared with older patients (16, 28, 31 years) investigated by Takanashi et al. [17]. It will be interesting to investigate the sisters when they get older.

PMG is a new neuroradiological finding in both our cases as it has been never reported in association with 4H leukodystrophy. It is our opinion that the PMG in these girls is most likely not due to the *POLR3B* mutations. Indeed, Wolf et al. has reviewed over 100 cases of POLR3-related leukodystrophy and has never encountered another case with PMG. However, since subtle PMG may be difficult to detect on MRI, it needs meticulous reading and is better seen with MRI protocols not typically performed in leukodystrophy patients, it is not possible to completely rule out that mutations in *POLR3B* are also causative for the PMG [17, 21]. PMG can be due either to genetic aberrations or to other causes (e.g., infections such as cytomegalovirus (CMV)). PMG has not been described in association with *POLR3* mutations in a paper published by Bizotto et al. [30] in 2015. We, therefore, suspect that patients have mutations in two different genes *(POLR3B* and another gene causing PMG*)*.

Both girls were hypotonic at birth, suffer from microcephaly and their developmental delay was observed during the 1st year of life. However, other neurological symptoms, such as ataxia and very intense tremors occurred later (Table 1). The age of onset ranged from 1 to 13 years in papers published by Bernard et al. and was estimated to be below 4–5 years in cases published by Terao et al. [12, 15]. Severe clinical symptoms and neurological deterioration were present, and developed so early in childhood that the older sister had to use a wheelchair at the age of 8 years. The younger patient could still walk at the age of 6 years despite progressive gait disturbances. The same observation was made by Tetreault et al. [13], who reported three European patients with *POLR3B* mutation presenting with mild developmental delay in early childhood and developed dysarthria as well as progressive motor dysfunction, including cerebellar ataxia. In all, two of these three patients showed progressive spasticity. In an article previously published by Saitsu et al. [8], the patient began to walk unsteadily at the age of 11 months, but retained her ability to walk as a teenager. In a paper published by Takanashi et al. [17] three patients with *POLR3B* mutations aged 16, 28, and 31 years could walk. In the multinational cross-sectional observational study published by Wolf et al. [21] in 2014, wheelchair dependence appeared at a mean age of 14 years. Only three patients with cataracts have been described to date [21, 32]. From a clinical point of view, our observations concerning cataracts are very important. Both sisters’ cataracts developed very rapidly. The cause of such rapid cataract development has not been yet identified. Furthermore, as our patients are hypothesized to have mutations in two different genes, it is unclear whether the cataracts are caused by the *POLR3B* mutations or are rather caused by the second gene mutation(s) leading to PMG, microcephaly, and dysmorphic features. Our female patients demonstrated dental abnormalities very typical for 4H leukodystrophy. Wolf et al. [21] described dental abnormalities in 87 % of patients, the majority (72 %) presenting with hypodontia. Our sisters did not have neonatal teeth, but they do suffer from hypodontia. Hypodontia and/or hypogonadism are not always present, as reported in previous articles [3, 8]. In 4H leukodystrophy developmental delay was noted in half of cases (52 % according to the data published by Wolf et al. [21]) and intellectual disability is also very common. We noted developmental delay in our patients at the age of 1 year and intellectual disability at 4 years. These two Polish girls presented with characteristic cerebellar features, such as ataxia and severe tremors. Pyramidal signs were not observed. However, our patients are young and, according to Wolf et al. [21], pyramidal signs are usually absent in young children. We did not observe epileptic seizures, although the presence of PMG and the abnormal EEG pattern observed, clearly indicate that they are at risk. Epilepsy is not a characteristic feature of 4H leukodystrophy; it was noted in 19 % of the patients [21]. Musculoskeletal signs are sometimes observed in 4 H leukodystrophy, such as hip dysplasia and flat valgus feet, as seen in our patients. At their last evaluations, both patients had (at the age of 11 years and 6 years) short statures and low weights (endocrine tests are normal in the older sibling), but hypogonadotropic hypogonadism as well as delayed puberty will be monitored when the time comes even if hypogonadotropic hypogonadism is sometimes absent. Indeed, delayed puberty was found in 69 % of the patients with *POLR3B* mutations [21].

## Conclusion

According to the literature, our observations, suggest that in young children without signs of hypogonadotropic hypogonadism, brain MRI pattern is essential for proper early diagnosis of 4H leukodystrophy. Although clinical and radiological characteristics are helpful, genetic confirmation is always necessary. Future studies will shed light on whether or not the PMG and cataracts are caused by the POLR3-related leukodystrophy or by another disorder.

### Conflict of Interest

On behalf of all authors, the corresponding author states that there is no conflict of interest.
